# The multivariate cox regression model for complete enteral nutrition after primary anastomosis in neonates with intestinal atresia

**DOI:** 10.3389/fped.2022.1071056

**Published:** 2022-12-12

**Authors:** Yang Chen, Le-dao Zhu, Ling Zhou, Ai-hui Guan, Zhi-yong Wang, Dong Xiao, Xiao-peng Ma, Feng Ren

**Affiliations:** ^1^ Shenzhen Children's Hospital, Shenzhen, China; ^2^ College of Medicine, Shantou University, Shantou, China

**Keywords:** intestinal atresia, cox regression, enteral nutrition, primary anastomosis, nomogram

## Abstract

**Objective:**

Enteral feeding after intestinal atresia has always been a concern for clinicians. But the present studies mainly focused on single factors. This research aimed to comprehensively analyze the multiple factors on complete enteral nutrition after primary anastomosis, and establish the convenient prediction model.

**Methods:**

We retrospectively collected reliable information in neonates with intestinal atresia form January 2010 to June 2022. The cox regression analysis was performed to select independent risk factors and develop nomogram. Subsequently, ROC curve, calibration curve and decision curve were drawn to thoroughly evaluate the accuracy and applicability of the model.

**Results:**

The predictors finally included in the model were gestational age, meconium peritonitis, distance from the anastomosis to the ileocecal region, diameter ratio of proximal to distal bowels, and time of initial feeding. The nomogram of predicting the probability of week 2, week 3 and week 4 was drawn and their area under the curve were 0.765, 0.785 and 0.747, respectively. Similarly, calibration and decision curve indicated that the prediction model had a great prediction performance.

**Conclusion:**

The clinical value of predictive models can be recognized. The hope is that the predictive model can help pediatricians reduce hospital costs and parental anxiety.

## Introduction

Intestinal atresia is a congenital disease characterized by the interruption of intestinal continuity. The main clinical manifestations are persistent vomiting, meconium missing and progressive abdominal distension after birth (1, 2). Complete intestinal obstruction can be resolved by immediate surgery, but oral feeding after operation will consume tremendous amount of hospital stay and cost, because children require continuous intravenous nutrition until full enteral feeding (3, 4). However, current studies mainly discussed the influence of the single factor on the recovery of postoperative intestinal function in children, which made it impossible for clinicians to comprehensively consider the prognosis. Therefore, by collecting information of neonates with intestinal atresia, this study expected establishing the clinical prediction model to investigate the risk factors for complete enteral nutrition after primary anastomosis.

## Patients and methods

### Patients selection

Inclusion criteria: newborns were diagnosed with intestinal atresia (jejunum and ileum) according to the intraoperative performance and postoperative pathology.

Exclusion criteria: incomplete case records; patients had other severe malformations such as gastroschisis and omphalocele; the patient developed serious postoperative complications, such as anastomotic leakage or stenosis; children suffered from serious systemic diseases such as sepsis or hypoxic ischemic encephalopathy during feeding; the operation was performed for enterostomy.

### Postoperative feeding strategy

The primary surgery of intestinal atresia is relatively onefold. The continuity of the bowel is reconstructed by removing the abnormal bowels and suturing the two broken ends. For the healing of the anastomosis and the recovery of intestinal function, regular and continuous intravenous nutrition should be used to meet the normal consumption and growth of newborns requiring prolonged fasting (2). According to the children's abdominal manifestations, exhaust and defecation, and abdominal x-ray findings, enteral nutrition will be implemented, and the intake should be gradually increased by observing the feeding tolerance. Significantly, nasal feeding can be temporarily applied for premature babies with poor swallowing function. When the children show feeding intolerance such as bloating and vomiting, pediatricians will avoid increasing the feeding amount, even abandon feeding again (3, 4). Finally, the terminus was identified when the patients were completely removed from parenteral nutrition. In addition, censored data was defined when full enteral nutrition had not been achieved before collection, and the children were withdrawn from feeding because of abandoning treatment or death.

### Information collection

This study was approved by Ethics Committee of Shenzhen Children's Hospital (No.202104202). Neonates with enterostomy were not considered because the continuity of bowels were not reestablished. Eventually, a total of 163 cases that met the requirements were retrospectively collected from January 2010 to June 2022 in Shenzhen Children's Hospital. The contents with high credibility were selected, such as gestational age, multifetation, gender, weight, preoperative examination, operation time, location of atresia, mesenteric angiodysplasia, proportion and number of anastomoses, time of first defecation, time of starting feeding, time to complete enteral nutrition, length of hospital stay and complications, etc. The classification of intestinal atresia is based on intestinal septum (type I), fibrous band (type II), mesenteric defect (type IIIa), mesenteric angiodysplasia (type IIIb) and multiple atresia (type IV).

### Statistical method

All data were statistically analyzed by SPSS20.0 and R software (version 3.34). Continuous variables satisfying normal distribution were expressed as mean (standard deviation), otherwise median (quartile) was applied. Moreover, categorical variables were represented as frequency (percentage). For further avoiding the potential bias caused by classification, stratified cox regression was applied in this study to eliminate the influence of confounding factors. Therefore, with complete enteral nutrition as the dependent variable, and pathological type as the stratification variable, all potential risk factors were initially screened by univariate COX regression analysis (*P* < 0.1). Then, the selected variables were subjected to multivariate COX stepwise regression. Meanwhile, visualized nomogram was drawn to facilitate the calculation of the predictive probability. Finally, the area under the curve (AUC) at different time point were calculated to evaluated the accuracy of the prediction model. The applicability of nomogram was assessed by the calibration and decision curve.

## Results

### General information

Based on inclusion and exclusion criteria, a total of 163 cases of intestinal atresia were included, of which 4 were interrupted because of treatment abandonment. All cases were divided into 5 groups according to pathological types, including 22 cases of type I, 17 cases of type II, 76 cases of type IIIa, 20 cases of type IIIb and 28 cases of type IV. The clinical characteristics of each group are shown in Table 1. There were no significant differences in gender, multifetation, preoperative blood examination, intestinal perforation, pulmonary infection, and diameter ratio of proximal to distal bowels (*P* > 0.05).

**Table 1:** General information of neonates with intestinal atresia after primary anastomosis.

Variates	I (*n* = 22)	II (*n* = 17)	IIIa (*n* = 76)	IIIb (*n* = 20)	IV (*n* = 28)	*P*
Gender/male	10 (45.45%)	10 (58.82%)	46 (60.53%)	9 (45%)	14 (50%)	0.571
Age of surgery/days	3 (2)	1 (1)	1.5 (1)	1.5 (1)	2 (2.25)	0.049
Gestational age/days	258.45 (17.75)	268 (10.67)	262.66 (15.81)	252.75 (13.59)	255.79 (14.95)	0.010
Multifetation	11 (50%)	7 (41.18%)	33 (43.42%)	6 (30%)	16 (57.14%)	0.267
Weight on admission/g	2648.64 (545.65)	2895.29 (444.58)	2840.39 (602.62)	2469.3 (494.03)	2550 (546.25)	0.019
Leukocyte ×10^9^	15.69 (7.59)	15.19 (6.12)	14.54 (7.8)	15.19 (9.69)	13.36 (8.62)	0.621
Hemoglobin/g/L	154.82 (20.73)	144.24 (22.3)	150.54 (23.65)	145.5 (24.18)	151.14 (21.81)	0.571
Albumin/g/L	32.7 (6.47)	32.1 (3.3)	33.75 (5.45)	32.3 (4.15)	31.75 (4.98)	0.608
C-reaction protein/mg/L	4.17 (4.61)	6.14 (6.4)	4.56 (5.36)	2.33 (7.21)	5.12 (7.72)	0.370
Operation time/min	95 (57)	128 (40)	120 (40)	120 (42)	143 (60.75)	<0.001
Meconium ileus	0 (0%)	0 (0%)	13 (17.11%)	6 (30%)	2 (7.14%)	0.011
Meconium peritonitis	0 (0%)	6 (35.29%)	25 (32.89%)	5 (25%)	4 (14.29%)	0.013
Intestinal perforation	0 (0%)	2 (11.76%)	11 (14.47%)	2 (10%)	1 (3.57%)	0.234
Pulmonary infection	1 (4.55%)	2 (11.76%)	8 (10.53%)	2 (10%)	4 (14.29%)	0.862
Mesenteric dysplasia	0 (0%)	2 (11.76%)	2 (2.63%)	20 (100%)	11 (39.29%)	<0.001
Anastomosis to ileocecal region/cm	40 (51.25)	30 (25)	40 (40)	62.5 (50)	50 (47.5)	0.047
Diameter ratio of bowels	7.25 (4)	7 (2)	6.84 (3.25)	7.5 (1.33)	8 (2)	0.157
Number of anastomotic	1 (0)	1 (0)	1 (0)	1 (0)	2 (3)	<0.001
Time of initial feeding/days	8 (2.75)	7 (3)	8.5 (5)	12 (7)	15 (10.5)	<0.001
Time of complete enteral nutrition/days	17.5 (4.75)	18 (5)	20.5 (15.25)	35.5 (19)	40.5 (36.25)	<0.001
Hospital stays/days	27.5 (16.75)	24 (9)	25.5 (17.25)	48 (25.5)	54 (36)	<0.001
Achieve full feeding	22 (100%)	17 (100%)	76 (100%)	20 (100%)	24 (85.7%)	-

### Univariate analysis

Univariate cox regression analysis was performed for all potential risk factors. The relationship between each variable and complete enteral nutrition was shown in Table 2. Age of surgery, multifetation, weight on admission, preoperative leukocytes, albumin and C-reaction protein, operation time, meconium ileus, intestinal perforation, pulmonary infection, mesenteric dysplasia, and number of anastomosis were initially excluded (*P* > 0.1).

**Table 2:** Univariate cox regression analysis for predicted factors.

Variates	*β*	*P*	Exp (B) (95% CI)
Gender	−0.543	0.008	0.581 (0.389–0.869)
Age of surgery	0.053	0.409	1.054 (0.930–1.196)
Gestational age	0.025	0.002	1.025 (1.009–1.042)
Multifetation	0.209	0.102	1.233 (0.960–1.583)
Weight on admission	0.000	0.531	1.000 (1.000–1.001)
Leukocyte	−0.003	0.851	0.997 (0.969–1.026)
Hemoglobin	0.008	0.049	1.008 (1.000–1.016)
Albumin	0.004	0.858	1.004 (0.963–1.046)
C-reaction protein	0.021	0.110	1.021 (0.995–1.047)
Operation time	0.001	0.518	1.001 (0.997–1.006)
Meconium ileus	−0.078	0.817	0.925 (0.478–1.790)
Meconium peritonitis	−1.005	0.001	0.366 (0.202–0.663)
Intestinal perforation	0.425	0.292	1.530 (0.693–3.377)
Pulmonary infection	0.368	0.218	1.444 (0.805–2.591)
Mesenteric dysplasia	−0.365	0.394	0.694 (0.300–1.608)
Anastomosis to ileocecal region	−0.011	0.001	0.989 (0.983–0.996)
Diameter ratio of bowels	−0.098	0.023	0.907 (0.834–0.986)
Number of anastomotic	0.045	0.65	1.046 (0.862–1.269)
Time of initial feeding	−0.058	<0.001	0.944 (0.915–0.974)

### Multivariate analysis

**Figure 1:**
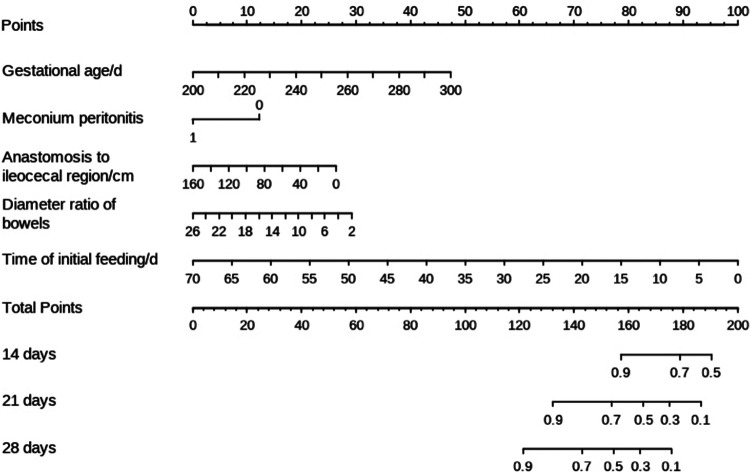
The nomogram for predicting complete enteral nutrition in week 2, week 3 and week 4.

The filtered variables were analyzed again by multivariate stepwise COX regression (forward: LR). The final independent risk factors included in the prediction model were gestational age, meconium peritonitis, distance from the anastomosis to the ileocecal region, diameter ratio of proximal to distal bowels, and time of initial feeding, as shown in Table 3. Meanwhile, the nomogram predicting the weekly probability of achieving complete enteral nutrition was plotted, based on the *β* coefficient and hazard ratio (Figure 1).

**Table 3:** Multivariate cox regression analysis for prediction model.

Variates	*β*	*P*	Exp (B) (95% CI)
Gestational age	0.025	<0.001	1.026 (1.013–1.038)
Meconium peritonitis	−0.744	0.001	0.475 (0.307–0.736)
Anastomosis to ileocecal region	−0.009	0.002	0.991 (0.985–0.997)
Diameter ratio of bowels	−0.091	0.024	0.913 (0.844–0.988)
Time of initial feeding	−0.065	<0.001	0.937 (0.910–0.966)

### Model evaluation

**Figure 2:**
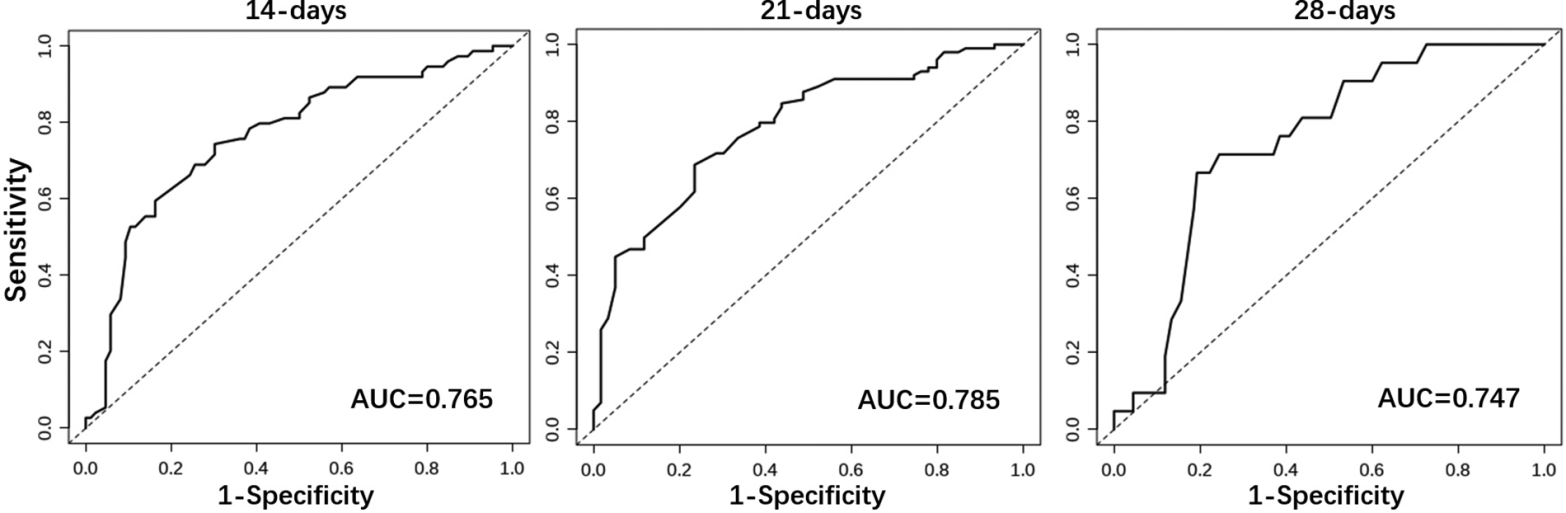
The ROC curves of prediction model in week 2, week 3 and week 4.

**Figure 3:**
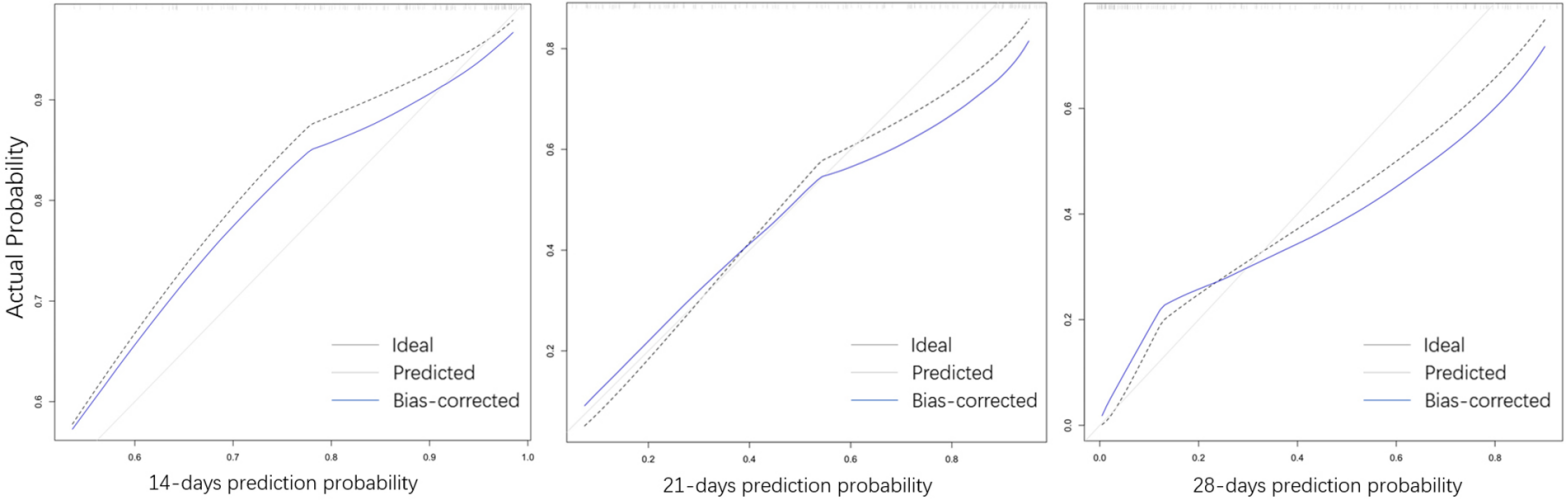
The calibration curves of the nomogram in week 2, week 3 and week 4.

**Figure 4:**
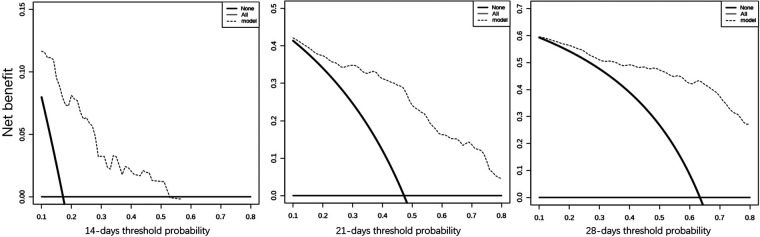
The decision curves of the nomogram in week 2, week 3 and week 4.

Considering the outcome metrics, the ROC curves were drawn at the time points of week 2, week 3 and week 4 (Figure 2). The areas under the curves of the three were 0.765, 0.785 and 0.747, respectively, which indicates that the accuracy of the model was satisfactory. Then, the calibration curve (Figure 3) drawn by multiple random overall sampling shows that the predicted curve fit well with the ideal curve, which proved that there was no overfitting performance of the prediction model. The decision curve (Figure 4) evaluating the predicted return suggested that the net benefit curve did not intersect the null line, revealing that patients can benefit from the predicted outcome.

## Discussion

With the development of prenatal ultrasound, more children with intestinal atresia are identified and treated early, and intestinal anastomosis has been quite skilled for qualified hospitals (5). Besides, mature intravenous nutrition can meet the needs of newborns for a long time. Therefore, the main current obsession for children with intestinal atresia is how to restore enteral nutrition as soon as possible. After all, only after the children get rid of intravenous nutrition can they be considered for discharge (6, 7). Lacking sufficient cases and detailed medical records, the existing studies only focused on the impact of a single factor. In this study, we retrospectively collected the clinical information of 163 children with intestinal atresia after primary anastomosis, so that multivariate analysis of risk factors affecting complete enteral nutrition can be implemented. We hoped that the prediction model can be useful for pediatrician's judgment and intervention, and prognostic inference can also promote communication with family members.

Statistical analysis showed that gestational age, meconium peritonitis, distance from anastomosis to ileocecal region, diameter ratio of proximal to distal bowels, and time of initial feeding were independent risk factors for complete enteral nutrition in children with intestinal atresia after primary anastomosis. For the convenience of computing, the nomogram that directly calculated the weekly probability of achieving enteral nutrition was drawn. Moreover, both calibration and decision curves indicated that the model had a good prediction effect. These results indicate that the clinical prediction model can meet the conditions of clinical application.

The influence of gestational age on feeding has been studied in many studies. Because of the incomplete swallowing function and gastrointestinal development, many infants without gastrointestinal diseases still relies on parenteral nutrition (8, 9). Especially for preterm infants with low birth weight, the feeding strategy will be more conservative in order to prevent neonatal necrotizing enterocolitis (10). In addition, meconium peritonitis is usually caused by perforation of the dilated proximal bowel. The feces entering the abdominal cavity not only greatly increases the chance of infection, but also results in intestinal damage and paralysis, both of which are not conducive to the recovery of the intestinal function. Under the circumstances, many surgeons give priority to enterostomy in the case of possible serious infection (11, 12). Furthermore, the interruption of intestinal continuity makes the distal intestinal tract smaller because of long-term disuse atrophy, while the proximal intestinal tract expands passively due to obstruction. The diameter ratio of proximal and distal intestinal tubes reflects the size of distal intestinal tubes, and the distance from the anastomotic site to the ileocecal region represents the length of the distal slender bowels, all of which impair the efficiency of excretion (13, 14). Many studies have shown that early feeding is beneficial for the recovery of gastrointestinal function. The secretion of hormones and the stimulation of digesta can contribute to peristalsis of the bowel and growth of intestinal villi. At the same time, earlier initiation of feeding leads to faster attainment of complete enteral nutrition, because clinicians tend to gradually increase feeding amounts (15).

There is no significant difference in the operation timing of intestinal atresia because complete intestinal obstruction usually requires emergency surgery (16). Moreover, in order to improve the surgical tolerance of children, surgeons will artificially add albumin and hemoglobin or prophylactic antibiotics, which reduces the impact of abnormal preoperative indicators (17). With the development of anastomotic techniques and materials, anastomotic leakage and stenosis have become minimal. The reason why the number of anastomosis was excluded may be that the increased number did not increase the probability of complications (18, 19). The short mesenteries can generate small and twisted bowels, and it also weaken the blood supply to the intestines. Intriguingly, mesangial dysplasia was ruled out in the initial screening. The possible explanation is that the determination of mesangial dysplasia depends on the subjective judgment of the surgeon, and the corresponding bowels are removed directly in severe cases, or even the treatment is given up (20).

Limited by the low incidence and long follow-up time, the accuracy of the model was only verified by internal cross-validation. Future multicenter studies need to be attempted because external validation can better illustrate the applicability and extrapolation of the prediction model. Meanwhile, the issue about feeding after enterostomy is worth exploring in the future.

In conclusion, this study has obvious reference value on enteral feeding after primary anastomosis of intestinal atresia. Pediatricians can evaluate and intervene timely to achieve compete enteral nutrition quickly and safely, according to the risk factors in the prediction model.

## Data Availability

The original contributions presented in the study are included in the article/**Supplementary Material**, further inquiries can be directed to the corresponding authors.
